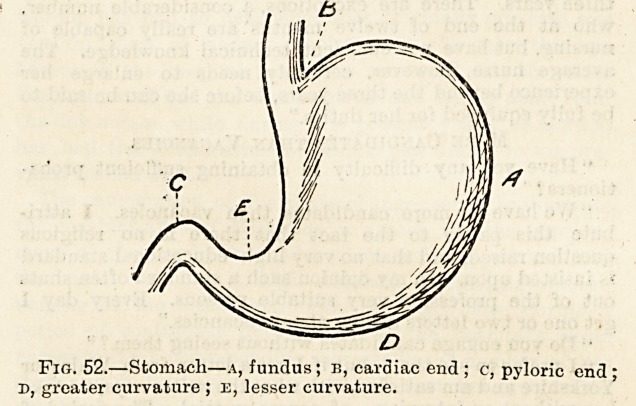# The Hospital. Nursing Section

**Published:** 1902-07-26

**Authors:** 


### The Hospital.

### Nursing Section.

Contributions for this Section of "The Hospital" should be addressed to the Editor, "The Hospital" Nursing Section, 28 & 29 Southampton Street, Strand, London, W.C.

No. 826.—Vol. XXXII. *SATURDAY, JULY 26, 1902.*

### Notes on News from the Nursing World.

### THE NEW MATRON OF ST. THOMAS'S HOSPITAL.

On Wednesday afternoon Miss Harriette E. G. Hamilton was appointed matron of St. Thomas's Hospital in succession to Miss L. M. Gordon, whose retirement we announced a few weeks ago. Miss Hamilton entered St. Thomas's Hospital in October, 1886, and became sister of Christian ward in March, 1888. In September, 1892, she was elected matron of Carlisle Infirmary, and in May, 1894, matron of the Victoria Hospital for Children, Chelsea. Five years later, in June, 1899, she was appointed matron of University College Hospital, Gower Street. Between 1892 and 1894 we believe that Miss Hamilton acted as assistant matron at St. Thomas's. She has therefore not only enjoyed the advantage of being at St. Thomas's for several years, but has also had valuable experience in other London hospitals and in the country. Under her auspices the entire system of nursing at University College Hospital has been revolutionised, and there is no question as to her qualifications for the extremely important post of head of the Nightingale School.

### NURSES FROM SOUTH AFRICA.

In future the terms "Army Nursing Service" and "Army Nursing Service Reserve" will be dropped, and all nurses in the employ of the War Office will be known as members of Queen Alexandra's Imperial Military Nursing Service. Sisters B. J. Talbot, and L. M. Fletcher, both time-expired, disembarked from the *Lismore Castle* last week. The *City of Vienna*, which arrived at Southampton on Saturday, had on board Sisters J. B. Robertson and J. E. Rogers, time-expired, and neither of them will return to South Africa. The *Dunottar Castle* arriving on the same day, had on board Sisters T. C. Sanders and L. M. Biggs, who do not return to South Africa owing to the reduction of the establishment. The *Tagus*, which arrived on Monday, had on board Sisters N. V. Blythe and B. M. Bentham, who, for the same reason, do not return to South Africa. The *Canada*, which left Capetown on July 8th and is due at Southampton on Friday or Saturday, has on board nursing Sisters K. S. MacQueen, E. Miller, E. H. Becher, E. M. McCarthy, C. McGowan, A. B. Smith, M. E. Greenham, and N. N. Hansom. The *Sicilia*, which is due on Wednesday next, has Sisters A. F. Hobbs and Crosby on board; the *Dunolly Castle*, due August 2, Sisters R. Gwyer and L. M. Searle; the *Colombian*, also due August 2, Sisters Fraser and McDonald; and the *Victorian*, Sisters Lee and Chitty. The *Staffordshire*, due August 3, has on board Sisters M. Simey and A. A. Knight; the *Malta*, due August 4, Sisters A. Guthrie and A. J. Moffat; the *Corinthian*, due August 6, Sisters A. M. Poulter, A. Rogers, A. Cleghorn, and F. Epton; and the *Pembroke Castle*, also due August 6, Sisters M. Careswell, M. L. Harris, and M. O. C. McCreery.

### PRIVATE NURSES IN RANGOON.

The folly of nurses rushing to Rangoon on the strength of reports that there is an opening for them in that town was pointed out by us in May, when we quoted the statement of a correspondent, and we subsequently emphasised the warning. This week it will be seen that a nurse who has had experience in Rangoon makes it clear that, in addition to a trying climate and minor discomforts, the hospital accommodation provided for various classes, the cost of living, the difficulty of obtaining lodgings, and in fact the general expenses, require to be taken into account. She agrees that midwives find plenty of employment, but her figures should be carefully studied and weighed by any nurse who thinks of going out to Burmah, even if she has a remunerative engagement offered her.

### THE MIDWIVES BILL.

On Monday evening the Lords' amendments to the Midwives Bill were considered in the House of Commons and agreed to. No objection was offered to the clause providing for the representation on the Midwives Board of one person appointed for a term of three years by Queen Victoria's Jubilee Institute for Nurses. At last, then, the authors of the measure may be congratulated upon succeeding, after many years, in steering it safely into port and getting a good deal more than they ventured to ask for.

### PREPARATORY TRAINING AT WALTHAM.

An interesting feature of the report of the Waltham Training School in the United States, which has been sent to us, is the description of the preparatory course. Established in 1894, the managers have now given this preparatory six months' training to fifteen successive classes of probationers. It is divided into six branches: the first, domestic science, embraces chemistry, dietetics, fermentation, putrefaction and decay, with special references to their effects on food, marketing and cooking. Housekeeping forms the second branch, and believing that "nursing is largely housekeeping for the sick" the Waltham School have all the housekeeping work of the home done by a probationer under the close supervision and constant teaching of the superintendent or her assistant. The class is divided into squads, each having its special part of the housework. As soon as the squads of probationers become proficient in the kind of work assigned to them, they are changed about, each to take up some new work. The third branch includes the instruction given in anatomy, physiology, medical chemistry, and bacteriology. The fourth, which is characterised as the most valuable of all, is district visiting and nursing of infants, convalescents, and chronic patients. Here, too, the class of probationers is divided into squads in order that each in turn may have practice in every branch of the work. "Personal improvement" forms the fifth branch. In addition to six lectures on the history of nursing and "its high meaning," four lectures are given upon personal hygiene, eight lessons in note-taking, four in clinical records, and eight in reading aloud. Once a week, for several months, there is a class in voice culture, and there are also regular gymnastic exercises under the direction of the instructor in physical culture. The sixth branch is the care of the outside of the body, or surface nursing. For two hours, three times a week, for three months, thorough instruction is given, and skilful work required, in massage, in manicuring, and in the care of the scalp and hair. The probationers practise upon each other, upon the junior and senior students, and upon patients able to come into the classroom of this department, which is fitted with all possible material and conveniences for the work. At the end of the six months probationers who have passed the examination, and "have in every way proved themselves fitted," are allowed to enter upon their regular three years' course.

### OUT-DOOR UNIFORM.

Last week a contributor who wears out-door uniform, but apparently would much rather not wear it, made some practical suggestions for the benefit of nurses who are in the same position as herself. It will be seen from our report of an interview with the matron of Wandsworth and Clapham Union Infirmary—which so far as nursing is concerned is well to the front, though the Wandsworth Board of Guardians would do well to prevent the adult wards from being over-run by children—that out-door uniform is compulsory for the nurses of that institution. The matron's view is that the uniform should stamp the institution, but in order to accomplish that end, she thinks that some kind of legal recognition is required to hinder it from being worn by unauthorised persons. We share her regret that the uniform of a nurse should be abused, but we are sure that she is right in doubting whether a majority of matrons would care to join in a movement for obtaining State protection. There is no enactment against the wearing of clerical attire by men not in holy orders, nor against the wearing of wigs by men who have not been called to the bar; and it is better that nurses should rely upon the trend of public opinion to save their uniform from discredit, than that they should appeal to the arm of the State.

### NURSES FOR SCHOOL CHILDREN.

At a drawing-room meeting held last week in support of the London School Nurses' Society, Lady Windsor in the chair, the annual report was considered. Mr. Brudenell Carter, in moving its adoption, stated that during the past year there had been a slight increase both in the money received and in the work undertaken by the society; but, inasmuch as the subscriptions only amounted to about £180, and it was calculated that £500 a year would be necessary to supply nurses to all the poorer London schools, the present state of affairs was not considered satisfactory. The society's nurses, he pointed out, attended the schools at regular intervals, and cleansed and healed the sores and wounds of the children. In the great majority of cases the parents as well as the children thoroughly appreciated what was done for them. Mrs. Homan seconded the motion, and it being agreed to, Mr. Lyulph Stanley moved a vote of thanks to Lady Windsor. He pleaded as the reason for obtaining an income of £500 that the society could then afford to employ nine or ten nurses.

### NEGLECTED WORKHOUSE CHILDREN.

At the meeting of the Leigh Board of Guardians last week a communication was read from the Local Government Board stating that, after fully considering the circumstances which led to the inquiry concerning the condition of the Leigh Workhouse children upon their return to Leigh from the Roman Catholic certified schools at Holly Mount, Tottington, and having regard to the circumstances disclosed at the inquiry, the Board are of opinion that it was established that the condition of the children changed for the worse during their residence at the school, and that they were sent back to the workhouse in an unsatisfactory state both as regards health and cleanliness. The Board exonerates the sisters from wilful neglect, but affirm that there was at the institution an absence of proper administration, which led to very unfortunate results, and that the conditions at Holly Mount fall short in many important respects of the standard required in poor law schools. In these circumstances we are not surprised that the Board have directed the withdrawal of the school certificate for three months. The incident suggests that there is scope in the schools certified by the Local Government Board for the visits of a trained nurse.

### NORFOLK TRAINED NURSES.

The thirty-fifth annual report of the Norfolk and Norwich Staff of Nurses has iust been issued by the committee. It is stated that the demand for nurses increases every year, and that the staff now consists of 41 surgical, medical, and monthly nurses. There are also four probationers in course of training at St. Mary's Hospital, Paddington, and the Sheffield Royal Hospital. The committee lately decided upon a new departure, and in future a small number of competent district nurses will reside together in a house which has been taken for the purpose in order to supply the needs of the poor who can afford to make a small payment. It has also been determined to co-operate in the work of the County Nursing Federation by receiving their probationers for a short period in order to test their capabilities, and to give them some insight into cottage nursing. Not the least interesting feature of the report is contained in the announcement that the committee, feeling that long and faithful services should have a special reward, have arranged to offer to those of their elder nurses who have rendered such service for at least ten years, and who are considered by the lady superintendent worthy of such recognition, leave of absence and sufficient pecuniary assistance to enable them either to take some additional special training or an entire rest for two months. The financial statement shows that the nurses earned the handsome amount of £2,477.

### THE NORTHAMPTON GARDEN FETE.

This is the time of year for outdoor entertainments on behalf of nursing organisations in need. We hope that the success achieved at Northampton, where a garden fête has just been given in order to assist the District Nurses' Fund, may prompt people in other towns to a similar effort. The fête consisted of tennis, croquet, and ping-pong tournaments, with a sale of work. The latter was the result of the labours of the Queen's Nurses' Needlework Guild, which consists of about a hundred members, who are interested in the work of the nurses. The quantity and quality of the fancy articles and needlework—not to mention the marmalade and chutney made by the nurses themselves—sold at the stalls afforded a practical proof of energy and devotion. Credit must also be given to Mr. John Cooper, chairman of the house committee of the fund, for his kindness in permitting the gathering to take place in Delapre Park, which is admirably suited for the purpose.

### PICNIC AT YORK.

On Thursday last week, owing to the kindness of Dr. Anderson and Mr. Spence, several of the sisters and nurses of the County Hospital, York, had a delightful run up the river Ouse in Mr. Spence's steam launch as far as Linton Locks. It was an ideal day for a picnic, and the nurses entirely forgot the cares of the hospital in the enjoyment of the sunshine and breeze, not to speak of skipping, tug of war, and swinging, which found much favour during the pleasant trip.

### THE RELIGIOUS QUESTION AT ARMAGH.

The Armagh Board of Guardians continue apparently to judge of the capacity of a nurse by her theology. They have appointed a nurse for the fever hospital, but when the vote was given as to the merits of two selected candidates twenty-six Protestants voted for one, and eight Romanists for the other. Subsequently, they had also to elect a midwife for the Richmond Dispensary District, and in this case, too, all the Protestants avowedly voted for a candidate who declared herself a member of the "Church of Ireland." So long as the Armagh Guardians allow their religious views to govern them in making appointments which ought to be obtained purely on professional fitness and personal character, they will deserve to encounter difficulties in filling up vacancies satisfactorily.

### THE TOTAL ABSTINENCE LEAGUE.

By the kindness of Lady de Rothschild the members of the Nurses' National Total Abstinence League were entertained at 9 Grosvenor Place last Friday afternoon. They were received by Lady de Rothschild and Lady Battersea. A delightful programme of music and recitations, arranged by Miss K. Dillon, the honorary secretary of the League, was performed, and a lecture by Dr. Sims Woodhead on the advantages of total abstinence from the nursing point of view was delivered. Dr. Woodhead said that "after much scientific research and pathological study he had come to the conclusion that nurses do not require alcohol; that by their practice and teaching of total abstinence they might help someone who is in danger from taking alcohol; and that they might be able to rescue others who are rapidly succumbing to its baneful effects." Nurse Butcher gave expression to the satisfaction with which the members of the League had listened to the address, and the hostess and Lady Battersea assured Dr. Woodhead of their deep appreciation of his kindness in sparing time to urge the cause of the League. Several new members were proposed.

### BLACKBURN NURSING ASSOCIATION.

The staff of the Blackburn Nursing Association consists of a superintendent and seven nurses, but it is affirmed in the local papers that if this number were doubled to-morrow "there would not be one nurse too many." The inevitable inference is that either the existing nurses are sadly overworked, or that the sick poor in the town are often neglected. The number of cases nursed last year was 1,036, but the number of visits paid is not stated. One of the nurses was specially engaged for patients who are willing to pay, but this is only an experiment. Unless the experiment answers, the scheme will be abandoned. As we have often contended, district work should, if possible, be kept entirely separate from the work of nursing paying patients. This cannot, perhaps, always be done, but we question whether if the Blackburn Association are relying upon a doubtful innovation, introduced after five years of excellent results, to augment the funds the object will be achieved. Many people who will subscribe to a movement wholly intended to help the sick poor in their distress will not contribute to an organisation which receives fees from fairly well-to-do patients. The extension of the work at Blackburn, which is clearly called for, is more likely to be accomplished by steady, persistent appeals to the residents than by the addition of a private nursing branch.

### STEADY PROGRESS AT ACCRINGTON.

The proceedings at the annual meeting of the Accrington District Nursing Association were, not without reason, of a congratulatory character. Every year appears to bring greater prosperity to the society, the twelve months just ended being the most satisfactory period it has passed through. The visits of nurses increased from 200 per month to 600, making a total of 6,485 for the year, and the receipts exceeded the expenditure by the handsome sum of £75. As the endowment fund of the association—which is invested with the Accrington District Gas and Works Board—now amounts to £1,150, the proposal to make a further addition to the nursing staff is quite justified. Dr. Ruttle, who testified in the most cordial terms to the excellent work done by the nurses, suggested that in the event of another nurse being appointed she should be entirely set apart for dealing with infectious cases. Assuming that he means infectious diseases which can be treated in the homes of the patients, such as measles and chicken-pox, the idea is an excellent one. A proposal that a nurse should be deputed to examine the eyesight of school children was also made, but Dr. Ruttle pointed out that such a nurse would require to have had a special course of training.

### THE QUEEN'S NURSE AT LIMERICK.

The Limerick Nursing Association has now been in existence for five years, and the report adopted at the annual meeting indicates that it is established on a firm basis. The number of cases nursed last year was 153, and the number of visits paid was 2,560. The amount received from subscriptions was £140, or £5 less than the cost of the work of the association. The balance was paid out of the amount derived from invested capital. It is a pity that this should have been necessary, but as the credit balance is increasing each year, the appropriation of a few pounds for ordinary expenses is not serious. The more important point is the evidence which the situation at Limerick affords of the success with which, by judicious management, the Irish branches of the Queen Victoria Jubilee Institute can be carried on.

### Lectures to Nurses on Anatomy.

By W. JOHNSON SMITH, F.R.C.S., Principal Medical Officer, Seamen's Hospital, Greenwich.

### LECTURE XXII.—THE ORGANS OF DIGESTION.

THE organs which digest food and thus render its nutritive parts fit to be taken up by the different structures of the system, consist of a long *alimentary tube* extending from the lips to the end of the lower bowel, and *accessory glands* varying in size and structure, the secretions of which, when discharged into the canal, effect chemical and physical changes in the food.

Instances of the accessory glands are met with at the very beginning of the alimentary canal, in the three pairs of *salivary glands*; the *parotid* below the ear and at the back of the cheek; the *submaxillary* at the upper part of the neck and behind the lower jaw, and the *sublingual*, as its name implies, below the front part of the tongue. The parotid, although the largest of these glands, cannot well be made out on the surface when healthy, but when inflamed and swollen, as in mumps, presents a conspicuous swelling. The salivary glands do not all secrete the same kind of fluid, but the different secretions when mixed together make up a clear and slightly viscid or sticky fluid which collects in the mouth, not only during a meal, but also, as the expression "making one's mouth water" implies, at the very idea of one. Insalivation, as the function of these glands is termed, is essential to good digestion. We all know how difficult it is to swallow food when "the mouth is dry," in illness or from mental emotion. The saliva softens solid food, and so assists the cutting and grinding action of the teeth; it lubricates the mass so that it can be readily swallowed, and by separating the particles of the food it assists taste. Moreover, by its chemical properties, it takes a preliminary part in the important digestive process of converting the starchy constituents of food into grape sugar.

The food, after it has been broken down by the teeth, and softened and partly dissolved by the saliva, is forced by the tongue and cheeks into a large cavity at the back of the mouth and nose termed the pharynx, the entrance to which is guarded in front by the soft palate and the uvula, and at the sides by the two tonsils. This cavity may be regarded as a large ante-chamber to both the air-passages and the alimentary canal, and there is on either side a long narrow passage—*Eustachian tube*—between it and the internal ear. It has free communication in front with the nostril as well as with the mouth. It is lined by mucous membrane which is frequently the seat of growths known as adenoids. The existence of this membrane, when inflamed, is made known to us by "hospital" and other forms of "sore throat." The sym athy of ear with throat in both acute and more or less ndolent affections of the pharynx may be explained by the communication kept up between these two parts by the Eustachian tubes.

By the voluntary or will-controlled action of three muscles called constructors the food is forced into the *œsrphagus* or gullet (fig. 51, *œ*), a closed but extensible tube about ten inches in length, and provided with an inner lining of mucous membrane which extends, between the spine behind and the trachea in front, from the pharynx to the stomach, passing from the thorax to the abdomen through a special opening in the diaphragm. The food which on reaching the œsophagus has passed beyond the control of the will, is propelled towards the stomach by successive waves of muscular contraction. The coats of the œsophagus are almost wholly comprised of muscular fibres, some arranged in lorgitudinal, others in circular bands.

In following the gullet through its opening in the diaphragm we pass into the abdomen, the largest of the three main cavities of the hnman body, which contains, in addition to the long and tortuous alimentary canal, the solid and glandular organs of digestion, the spleen and the two kidneys. To these should be added, as the so-called pelvic cavity really forms a part of the abdomen, the urinary bladder and the organs of generation. Although all these organs, like those of the chest, are closely packed together, considerable enlargement of any one of them or rapid and extreme distension of the intestines is permitted by the yielding of the soft and muscular walls of the cavity, which thus differ from the rigid framework of the chest and the compact and still more rigid brain-case. In the upper part of the abdomen, protected by the lower ribs and in contact with the diaphragm, are the liver and the spleen (fig. 51, *l*, *sp*),

FIG. 51.—The Alimentary Tract—*c s*, fundus of stomach; *s*, greater curvature of stomach; *œ*, œsophagus; *s p*, spleen; *d*, duodenum; *i*, small intestines; *l*, liver; *g b*, gall bladder, *v a*, vermiform appendix; *c*, cæcum; *a c*, ascending colon; *t c*, transverse colon; *d c*, descending colon; *i c*, sigmoid flexure; *r*, rectum; *p y*, pylore end, and *p*, pylorus of stomach.

the former on the right side, and the latter which, when healthy, is a much smaller organ, on the left. Below and behind the left portion of the liver and in front of the spleen is the stomach which for the most part is placed on the left side (fig. 51, *a*, *cs*). Extending across the front of the spine and behind the stomach is the pancreas. The two kidneys are placed at the back of the cavity under cover of the floating ribs. The bladder lies behind the front of the pelvic girdle, and, in women, in front of the organs of generation. The rest of the abdominal cavity is occupied by intestines and by the thick and large layer of fat —the omentum—by which the intestines are covered.

The cavity containing these different organs like that

### LECTURES TO NURSES ON ANATOMY.—*Continued.*

containing each of the lungs is furnished throughout by a thin serous membrane which lines the inner surface of its walls and is reflected over its contents in the same way that the pleura is continued over each lung. This thin membrane, known as the peritoneum, is a tissue of great importance from a surgical as well as from an anatomical point of view, as inflammation so readily excited by the contact of foreign and more or less septic material admitted either by an external wound, by rupture of intestine, or the opening into the abdomen of an internal abscess, constitutes one of the most fatal and most painful of acute diseases. This membrane is reflected at once over some organs, the kidneys for instance, and maintains them fixed and in close contact with the abdominal wall. In other organs it forms a broad and short ligament; and in others again, as in the small intestines, it forms a long and broad membrane called the mesentery.

The stomach in which the food previously converted in the mouth into a pulp is churned into a thick fluid called chyme is an expanded muscular bag, situated, as we have already learnt, in the upper part, and chiefly on the left side of the abdominal cavity. This organ varies much in position, size and form, moving with every act of breathing, and capable when much distended by food and gas of filling up a considerable portion of the abdomen and descending almost as far as the pelvis. When empty it is much contracted and hidden behind the left half of the liver. These wide variations in size are associated with equally marked changes in the shape and direction of the organ.

The stomach is much expanded on the left side, where it presents the *fundus* (fig. 52, A), and contracted on the right side towards the commencement of the intestine, where it forms the pyloric extremity. At its upper margin to the right of the fundus the œsophagus opens into the stomach by what is known as the *cardiac orifice* (B). The tapering portion of the organ communicates on the left side with the upper end of the intestinal canal by a constricted opening surrounded by a well-marked and thick

Fig. 52.—Stomach—A, fundus; B, cardiac end; C, pyloric end; D, greater curvature; E, lesser curvature.

ring of muscular structure, which is called the *pyloric orifice* (C). The long curved lower border of the stomach extending from the left side of the cardiac orifice to the lower part of the pylorus is called the *greater curvature* (D), and the much shorter border extending between these two orifices above the *lesser curvature* (E).

### The Nurses of Wandsworth and Clapham Union Infirmary.

INTERVIEW WITH THE MATRON: BY OUR OWN COMMISSIONER.

The Nurses' Home at the Wandsworth and Clapham Union Infirmary is one of the most commodious and comfortable, as well as one of the most handsomely equipped, in the United Kingdom. The general sitting-room is very large, is furnished both tastefully and well; there are separate sitting-rooms for the sisters; each nurse has a bedroom of excellent size, with every possible convenience, to herself; the bathroom accommodation is adequate; there is a covered way from the infirmary to the home; and altogether the institution, from every standpoint, is thoroughly up-to-date and on a line with the leading London hospitals.

"But," as I said to the matron, when we had concluded our inspection of the charming home, "where do your nurses dine?"

"The mess-room," Miss Anstey replied, "is quite distinct from both the infirmary and the home. We think it better that no food should be served in the home, except in case of illness. And all the meals of the day nurses are taken in the mess-room."

Subsequently, I saw the nurses at tea in the mess-room, which is large and airy, and has the advantage of being in close proximity to a very fine kitchen. The service of the meals is thus rendered exceedingly easy.

As the matron proceeded to say, "Even before the home, with its 73 bedrooms was opened in 1900, the majority of the nurses had a bedroom to themselves. But the provision was not adequate, and the guardians decided upon the erection of the present building, which is in charge of the home sister."

Nor can it be alleged that while the nurses are well cared for, the patients are neglected. If the exterior of the infirmary, which is now undergoing alterations in order to enlarge the receiving rooms and offices, is not elegant, the wards are capacious and lofty with all the necessary provisions. In fact, the only feature that cannot be commended is the matron's office in the infirmary, which is small, dark, and, even in summer, dreary. Not that she complained of the accommodation, and on the afternoon of one of the hottest days in the year, the room was beautifully cool.

### Male Attendants.

"How large is your staff at the present time?" I inquired.

"There are sixty-seven female and four male attendants."

"What are the duties of the male attendants?"

"They are in attendance on the male lunatics, of whom we get a considerable number. Eight years ago we only had one male attendant, but we have found it a decided advantage to have men to look after the male lunatics. Some of the patients are very difficult to deal with, and we think that it is altogether more proper to have male attendants for them. All the male patients are bathed by male attendants. This has always been the rule since the formation of the training school.

### Foundation of the School.

"Was the training school established in your time?"

"I am the first trained matron of the infirmary, but the training had been put on foot three years before I came, which was in 1887. My predecessor was not trained, but the present medical superintendent, Dr. Breward Neal, established the teaching and training of nurses in 1884.

"You did not start with three years' training?"

"No, with two years. The term was extended to three years in 1896. The certificate is usually earned at the end of two years, but is not given until the expiration of the third. Speaking from experience of both, I consider the three years' system better. It takes a probationer one year to understand what she has undertaken; the second year she gains the knowledge that enables her to perform the duties of a nurse and assures full responsibility; and in the third year

### THE NURSES OF WANDSWORTH AND CLAPHAM UNION INFIRMARY.—*Continued.*

I think she should put forth her knowledge and capabilities in order to repay the school for all the trouble that has been taken in her training, bearing in mind the fact that she pays no premium and receives a salary from the outset."

"Are you in favour of still further increasing the period of training?"

"I am not prepared to urge that probationers should be bound for four years. But I do not think that the average nurse is sufficiently experienced in nursing at the end of three years. There are exceptions, a considerable number, who at the end of twelve months are really capable of nursing, but have not sufficient technical knowledge. The average nurse, however, certainly needs to enlarge her experience beyond the three years, before she can be said to be fully equipped for her duties."

### More Candidates than Vacancies.

"Have you any difficulty in obtaining sufficient probationers?"

"We have far more candidates than vacancies. I attribute this partly to the fact that there is no religious question raised, and that no very high educational standard is insisted upon. In my opinion such a standard often shuts out of the profession very suitable persons. Every day I get one or two letters asking about vacancies."

"Do you engage candidates without seeing them?"

"I prefer to see them, but if I get a letter from Wales or Yorkshire and am satisfied with what the writer says, we take her without an interview—of course on trial. The period of trial is one, two, or three months, and during that time the arrangement can be terminated by a week's notice on either side. As a matter of fact, the candidates who have been engaged by letter have invariably remained for the full term of training."

"When you are choosing a probationer, what do you regard as the most essential qualifications?"

"Good health, sympathy, tact, and kindness of heart."

"As to the lectures," continued the matron, "there is a course every winter by the medical superintendent and assistant officer—sometimes the junior also lectures. The matron, assistant matron, and home sister give class instruction, and the sisters teach the ward work. There is an examination every year, which must be passed before the certificate is given. As a rule all the nurses pass, but if one fails she has the option of staying on a fourth year and trying again."

### Outdoor Uniform.

"What salary is paid in the first year?"

"Ten pounds, and in the second and third £18. Both indoor and outdoor uniforms are provided."

"Is the wearing of outdoor uniform compulsory?"

"Yes, except special permission is given, as in the case of cyclists who want to wear private dress. I make the concession when I can. But the uniform is there, and it must, as a rule, be worn. I am sorry to see that the uniform of a nurse is so much abused, and I should like to obtain for it some kind of legal recognition. A Local Government Board inspector once asked me if the matrons could not combine in favour of such a movement, but I doubted whether we should get a majority in favour. My view is that the uniform should stamp the institution; but it is so easily copied under present conditions, that all sorts of people wear it."

### Night Duty.

"Your official name for the sisters is charge nurses?"

"Yes; but they are generally spoken of as sisters. The hours of duty are pretty much the same for all grades, and so are the hours for meals, except, of course, the night nurses."

"When do the night nurses dine?"

"At 5.30 P.M. They have also two meals in the night, arranged at different times. The latter are partaken of in the sculleries attached to the wards. The rule is for the nurses to take four months' night duty and eight months' day duty. But sometimes I find it requisite to take nurses off night duty at the end of a week or two. So few persons can turn night into day, and the work is more wearing. I think it is very important that young nurses should not be kept too long on night duty, and that they should be taken off at the first evidence of breaking down. They should get accustomed to the extra strain by degrees."

"Have you any nurses who find night work suit them better than day work?"

"Fortunately, I have; and if there is a difficulty in changing a nurse I keep one of those who can stand the work, and indeed prefer it, in longer than the four months. But the allocation of night duty requires much thought and attention."

"How often are the night nurses off duty?"

"Two nights in each month. They also get an hour in the morning and an hour in the evening before going on duty. The day nurses have a day and half a day off each month, and they are at liberty every day from 7 until 10. The hours of the sisters, staff nurses, and probationers are the same. But as to holidays, the sisters have a month each, while the nurses have a week's holiday at the end of six months' probation, two weeks at the end of a year, and three weeks at the end of two years."

### Children in the Adult Wards.

"What are the arrangements in the wards?"

"There are 622 beds. But we have had 800 patients in the infirmary. The wards have either 32 or 28 beds each. On the male side there are two wards, a day room and a special room on each flat; and on the female three wards, a day room and a special. There are two probationers, and a sister on duty in every flat during the day, and a probationer in every ward during the night. The night superintendent is in charge of the whole building, but no sister is on duty in the night."

"How many children are you certified for?"

"Thirty-two. But we have had upwards of 150 in the wards. I allow an additional probationer to a certain proportion of children, and when the children are numerous there are, therefore, more probationers on duty on some flats than on others. The number of children is not a good thing for some of the adult patients and tends to retard their recovery. We keep them as much as possible in the day rooms, but even then the wards are not as quiet and comfortable as we should like them to be."

### The Value of Recreation.

"Is there anything in the shape of a library for the nurses?"

"They can have books from the chaplain's library when they wish. As you observed, there is a piano in the general sitting-room, in which, also, concerts are given. There is a tennis court, and every year the nurses have a dance, but not at the infirmary. Last year it took place in Wandsworth Town Hall. I believe in recreation as well as in strict attention to duty, and I am sure that nurses who cease to think of their duty when they are enjoying themselves take all the more interest in it when they return to the wards."

"Do your nurses ever stay on after they receive their certificate?"

"I never advise them to stay longer than four years, because I think that they should gain varied experience while they are young. As a rule they go from here either to private work, fever work, or district work. I have sent several to the South London Nursing Association and have received most satisfactory accounts of them from the matron. Some obtain the L.O.S certificate and take to midwifery. Others are Queen's Nurses—one is a matron. One of my nurses, after leaving here with merit, was sent to Maidstone at the time of the typhoid fever epidemic and earned the medal. She subsequently went to South Africa. Another nursed Princess Henry of Battenberg at Nice when she had a bad knee. These things quite repay me for any trouble I have in training, and are also practically a testimonial to the infirmary."

### A Matron's Primary Duty.

"I should be glad if you would tell me, in conclusion, what you esteem to be the most important duty of a matron in relation to her nurses."

"The study of individual character. Probationers are not always to blame, and sisters are not always faultless. I remember the time when I was a timid probationer, and I think that a matron should stand between probationers and sisters, and be as accessible to the one as to the other. Sisters are apt to have favourites, and it is the clear obligation of the matron to see that all her probationers are treated alike, and not to rely too much on one side or the other."

### The Retiring Matron of St. Thomas's Hospital.

The impending retirement of Miss L. M. Gordon from the post of Matron to St. Thomas's Hospital will be widely regretted, not only by the staff with which she has been so closely associated in work, but also by a wide circle of those who have been connected with the hospital during her term of office, and many in the profession of nursing elsewhere. Her resignation will be the more sympathised with, since it is due to the fact that Miss Gordon finds her health unequal to the strain of the further discharge of the onerous duties which she has so worthily performed during the past twelve years.

St. Thomas's Hospital has been well served in this office, as, since the migration to Lambeth, the post has, with the exception of a short period, been held by only two ladies. The first of these, Mrs. Wardroper, established the staff in its new home, and carried on a work with which her name will always be associated until June 1887. After a short period of two and a half years, during which Miss Pringle held office, Miss Gordon was appointed in March 1900, and has been the moving spirit in the many changes and improvements which have been introduced in her department until the present time.

Miss Gordon entered the Nightingale School as a probationer in September, 1874. After completing her course at St. Thomas's, and doing duty as temporary sister in the wards, she became assistant matron to the Liverpool Royal Infirmary, where she remained four and a half years. From Liverpool she proceeded to Leeds, where she held the important post of superintendent of the General Infirmary for a period of ten years, gaining golden opinions from those with whom she was associated.

Miss Gordon's appointment to St. Thomas's coincided in date with that of Mr. J. G. Wainwright, the present treasurer, and it is in conjunction with him that many important and far-reaching changes and developments, affecting both the interests of the patients and the comfort and well-being of the nursing staff, have been carried out.

The first important improvement during Miss Gordon's *régime* was the partitioning of the open dormitories formerly occupied by the nurses into separate bedrooms for each, and at the same time the existing linenry of the hospital was so subdivided as to provide a large day sitting-room for the nurses when off duty.

The next change was dependent on the substitution of female attendance in the operating theatres of the hospital for the time-honoured Surgery-man, and in conjunction with this alteration arrangements were made by which operations at night were attended by the night superintendent and thus the disturbance of the ward sisters' rest was obviated. The new system working well it was considerably developed and at present the staff in each theatre consists of a sister and two day nurses, one responsible for the instruments and one attendant, and one night theatre nurse. Beyond this the work of the ward sisters has been materially lessened as far as operations are concerned by the provision of all necessary dressings etc. in the theatres which previously had to be taken down on each occasion by the sister of the ward from which the patient was brought.

On the retirement of the late steward, Mr. Walker, a considerable change was made in the devolution of the main kitchen responsibilities and domestic arrangements from the steward to the matron. A special sister was appointed to supervise the staff and the department has since been worked in a highly satisfactory manner, the kitchen and offices being re-appointed and brought up to date so as to be able to undertake the whole of the cooking necessary for the patients, resident medical staff and nurses.

The number of Nightingale probationers has been increased from 35 to 52, this both augmenting the staff of the hospital and rendering recourse to outside assistance less frequent, and, at the same time, enabling all St. Thomas's nurses to obtain a full three years' training before taking up appointments elsewhere.

Accommodation for the constantly increasing nursing staff has been one of the most difficult problems with which Miss Gordon has had to deal, since that originally provided was very far short of modern requirements. To meet this two houses in the hospital have been converted into rooms for the night staff and the theatre sister and nurses apart from the day nurses, while during the present year Miss Gordon has had the gratification of seeing new dining-rooms provided for her staff.

The enumeration of the above changes serves to illustrate the constant aims of Miss Gordon's work, viz., the improvement of the conditions under which her staff worked, and the maintenance of a high standard of efficiency in the nursing of the institution. She has held office during a period of continuous development of the hospital's work, necessitating a corresponding increase in the number of her staff, and it is much to be deplored that her resignation should be necessitated at a moment when her most cherished project, the addition of a satisfactory home for the housing of the nurses, has become a matter of practical politics in view of the recent addition to the hospital's finances.

Beyond her work within St. Thomas's Miss Gordon has found time to help many kindred institutions and individuals with her advice and the fruit of long experience. In this connection it is especially to be regretted that the severance of her ties with the hospital necessitates also her retirement from the Board of Queen Alexandra's Imperial Nursing Service, where her ripe knowledge and sober judgment might have been of much service to the Army and the nation at large. Miss Gordon has been a member of the Committee of the Junius S. Morgan Benevolent Fund since its establishment, and has regularly attended the meetings where her presence has been much appreciated. Perhaps the most striking feature of Miss Gordon's powers of administration has been her constant exercise of tact, combined with a strong sense of justice. This characteristic it is which has allowed her to fulfil the duties of a difficult and responsible office efficiently and without a trace of friction with the many it has been her duty to work with and to satisfy.

Endowed with a spirit of enterprise, strength of purpose and excellent judgment, she has been both a progressive and successful matron, while her consideration for others and equable temperament have made her generally beloved by her own nurses, and by the medical staff of the hospital. Readily approached, none have hesitated to go to her for advice and sympathy with the full and well-founded conviction that she would give them the best she had, and that her active endeavours would be made both in the interests of the individual and the institution she represented.

Her friends can but wish her a long enjoyment of well-earned rest from an arduous and busy life, and that she may have the satisfaction of seeing her efforts further developed; meanwhile, she may rest assured of the respect and affection which her labours have inspired.

### Wants and Workers.

Referring to a note in The Hospital of June 28th headed "Nurses in the Hop-yard" a trained nurse writes to say that she would like to assist in such work, but could not afford to give time quite free. Does anyone require her services? E. D. 27 Shelgate Road, New Wandsworth, S.W.

### Nursing Diphtheria in a Girls' Home.

BY THE TEMPORARY MATRON.

As temporary matron of a home for girls in the North of England I have just had a rather unique experience, which, perhaps, may be of some interest to nurses in general. I had promised to take charge of the home during the absence of the matron, who was away on sick leave, and shortly after my arrival diphtheria broke out and fifteen of the twenty girls were attacked by it. How we met the situation, turned the home into a hospital, and then turned it into a home again during those three months, I propose to tell.

### The Home.

In the home there is accommodation for twenty homeless girls, varying in ages from four to sixteen, who are trained for domestic service. It is under the management of a local committee. The matron, save for the assistance of a kitchen matron, is single-handed. When I asked the secretary what would be done in the event of an outbreak of serious or infectious illness, he replied:—"We have been very fortunate so far, and have had only one case, diphtheria, which was isolated and nursed in the home." So I thought no more about such possible contingencies, and, although I found plenty of minor fleshy ills to attend to among the children, began to feel that a nursing qualification was not a *sine quâ non* for the matron of the home. I concentrated my attention in the direction of the daily round which is one that is rather difficult to describe. As I look back upon it now it seems a jumbled mass of loud bell-ringing early in the morning, a scuffle in dormitories, the sound of little feet hurrying to and from bath-rooms, and after that the noise of brushes and brooms. Then another bell, prayers, and breakfast, and the day's work had begun in earnest. For me a tour of inspection through the home, an interview and conspiracy with the kitchen matron in the store-room usually made a fair start. Next followed the correspondence and book-keeping and superintendence of the housework. I have hazy recollections of meals that were like perpetual Sunday-school treats, strange faces asking to be shown over the home, the sound of the children's voices as they romped and sang—how they sang, too, most beautifully—in the big play-room, and dismal moments when I had to read the Riot Act and enforce Spartan treatment. Memory recalls these reminiscences so faintly to my mind that I cannot realise that it all happened but a few months ago; because the only picture which stands out vividly is the picture of the home as a hospital during the diphtheria epidemic.

### The Outbreak.

One morning after the first few weeks of my reign one of the children was reported to have a headache and sore throat. I saw her, isolated her, and later in the day the doctor pronounced it to be diphtheria. We soon had another case, and still another, till we realised that a diphtheria epidemic was upon us.

The medical officer of the board of health said]he could arrange to have three patients sent to the isolation hospital, and the ambulance took them away. But each day brought fresh cases to light, and we had to set to work to turn the whole home into a hospital. Fortunately, they were all of a very mild type. We had no complications, and only the most simple, straightforward treatment was necessary. But still it was a very anxious time.

With the exception of the kitchen-matron—who thought it "new-fangled"—we all had anti-toxin, and this, no doubt, lessened the severity of the cases. For still they came, till at last we had only five girls who were not on the sick list. I had the help of a nurse who relieved me entirely of the night duty during the first week after her arrival, and subsequently had charge of all the patients until they were quite convalescent. Of course they were isolated, and every possible precaution in the way of disinfection was taken. But we could not remove the cause of the trouble: the opening up of an old drain during the making of a new one in the road in front of the home.

### The Children's Convalescence.

As the children became convalescent they were very amusing. Games of "Hospital," complete with doctor, matron, nurse, patients, charts, thermometers, throat brushes, and "feeds," were the order of the day. When each child was disinfected after quarantine and allowed to join the five who had not succumbed—from whom she received quite an ovation—experiences, graphically illustrated, were related, confidences exchanged, and every girl of the noble fifteen felt herself a heroine, an individual, no longer a mere inmate of the home. Towards the five who had endured the burden and heat of the day my sympathies were much extended, especially as they came in for a good deal of patronage from their more distinguished companions. One child, in vainly trying to impress a friend, who herself had kept out of the fray, with a description which the other could not quite follow, upon being asked to repeat herself replied haughtily, "I don't boil my cabbages twice for donkeys!" The nurse also heard another bit of repartee from the same girl upon a similar occasion: "Monkeys climb very high, but it takes fools to look at them!" I certainly think that on the whole the children got a good deal of excitement and distinction out of the epidemic, and after it was all over and things became fairly normal again, the usual monthly letter to their relations or patronesses contained a good deal about the "dipathetic" throats.

### Disinfecting.

The sanitary people were wonderfully obliging, meeting us in every possible way; and when the outbreak was really over, and the "thin red line" of five heroines had shown themselves to be diphtheria-proof, we were disinfected throughout. As soon as we were quite clean again, the local committee felt safe in coming near us once more, the girls gradually resumed their usual work—in reality being very little contaminated by their brief spell of spoiling—and the doctor and I shook hands and congratulated each other upon the happy issue of what might have been fraught with serious, even fatal results. And then, just as the home had begun to settle down to its normal condition, it was time for me to go!

### Presentations.

Plymouth Workhouse Infirmary.—Miss Nelly Nott, night sister, has just been presented with a pretty brass kettle, stand, and table vase, from the medical officer, superintendent, and nursing staff. She leaves on account of her approaching marriage, and carries with her many good wishes for her future happiness.

Aston Clinton Nursing Association.—Miss Lucie A. E. Saunders, who for the past 14 years has been working as Lady Rothschild's village nurse in the parishes of Aston Clinton and Buckland, Bucks, has lately been made the recipient of two very handsome presents from the inhabitants, on the occasion of her marriage with the Rev. Howell W. Jenkins, B.A. The presentation from Aston Clinton took the form of an enamelled brass clock with a pair of candlesticks to match, and that from Buckland of a silver-mounted oak salad bowl and servers. Upwards of two hundred subscribers testified to their gratitude for and appreciation of Miss Saunders' work amongst them.

### Everybody's Opinion.

**[Correspondence on all subjects is invited, but we cannot in any way be responsible for the opinions expressed by our correspondents. No communication can be entertained if the name and address of the correspondent are not given as a guarantee of good faith, but not necessarily for publication. All correspondents should write on one side of the paper only.]**

### A HOME FOR PRIVATE NURSES WANTED.

"M. F. R." writes from Dunkeld: I should like to say a few words concerning the Nurses' Hostel in Francis Street, so slightingly referred to by "A Private Nurse" in a recent number of The Hospital. I had the pleasure of staying for a short time at the new building quite recently, and I could not have believed (had I not seen for myself) that so much comfort and even luxury could be had for so modest an expenditure. "A Private Nurse" can have very little idea of what the running of such an establishment as the hostel means to the housekeeper, or indeed of the working of any such place, else she would not be so inconsiderate as to expect meals to be served at odd hours. I cannot properly express the kindly opinion I carried away of the Hostel and all its arrangements, and of its kind and capable sister superintendent.

### "TWENTY-FIVE VISITS A DAY."

"Nurse E." writes: I see from a note in The Hospital last week that a district nurse paid on an average twenty-five visits a day. I am a district nurse and find most of my visits take half-an-hour, some much longer. Many dressing cases (for example, cancer or chronic abscess), where there is also the patient to wash, hair to do, bed to make, take about one hour. Pneumonia cases where there is the patient to wash, etc., poultice, take temperature, pulse, and respiration, take about three-quarters of an hour, as do cases of acute rheumatism; subsequent visits during the day for pneumonia cases, where there is the poultice to change, temperature, etc., to take, about twenty minutes. Cases of paralysis and phthisis, about half an hour. I have worked in three different districts, and find in each the work chiefly consists of the above-mentioned cases. I certainly do not consider I am a slow worker, but I could not undertake to nurse my patients if I had twenty-five visits a day to pay. For the first three months of the present year I paid on an average ten visits a day, and found that quite as much as I could do to attend to my patients thoroughly, taking very little off-duty time. My district is scattered, but in fine weather I cycle, and in wet go by omnibus where possible.

### OUTDOOR UNIFORM.

"Nurse T." writes: In reply to an article in last week's issue of The Hospital regarding nurses wearing outdoor uniform, I should like to say that I have tried wearing both private dress and uniform, and find the latter affords a nurse much better protection from insults, etc. Also in travelling to a case one receives much more attention from porters and cabmen, as they seem to recognise the importance of a nurse's time. Then, too, one may be very useful in case of a street accident or sudden illness. For instance, a friend of mine was walking in a lonely country place, in outdoor uniform, when a man ran after her beseeching her to come to his wife, who was in a fit. On reaching the cottage she found the woman dangerously ill, and on the verge of her confinement, with no other help near than that of the patient's husband. She was able to render most valuable assistance. One thing I am sure of, namely, that there should be some distinction made between a nursemaid's uniform and that of a trained nurse, who, I think, should be able to show a certificate of proficiency, if this could possibly be brought into force. As to carrying germs into the ward I think if a nurse removes her apron, pins her skirt well off the ground, and buttons up her cloak, there should be no danger on that score.

"One who does not Wear it" writes: I do not see why nurses should wear uniform any more than doctors or lady doctors, except district nurses, who, of course, must do so in going from house to house. Nurses are as a rule very quick in their movements, and can change their dress in a very short time. I used outdoor uniform for seven years, I got very tired of wearing it, and sometimes felt ashamed of my uniform when I came across nurses looking very untidy. I was walking with a friend a short time ago, and we met half a dozen nurses who had been out for the afternoon. They all had bright, happy faces; their gloves and boots were unexceptionable; but, oh! their hair and their bonnets. I do not think anyone could say they looked neat or nice. I know many people who do not care to be seen about with a nurse in outdoor uniform. I remember a lady saying to me, "I am not going to walk with you: people will think I am your patient, and that it is a mental case." Then, again, so many children's nurses wear outdoor uniform now that it is difficult to know whether they are sick-nurses or children's nurses. At the Jubilee of our late Queen Victoria I was nursing some diphtheria cases in a cottage in a small village in Warwickshire. On the day of the event my patients were all so much better that I asked the doctor if I could go and look on at the rejoicings. He consented, and said he would go to the ground and see if the ladies objected to my going. While he was away I arranged with the mother of one of my patients—a boy of 14—to do duty for me: she had helped me with the night work. I put on my outdoor uniform, and was quite ready when the doctor returned. He said, "I am so sorry, nurse, but some of the ladies object to your going: one lady said, as soon as she saw the uniform, she and her friends would at once leave the ground, as they did not wish to get diphtheria." So the outdoor uniform had to come off again. I may add that I have often seen people cross over the road rather than pass me.

### PRIVATE NURSES FOR RANGOON.

"E. A. W." writes from Bangalore: A nurse who has seen even more of Rangoon and Burmah than I have has called my attention to two notes which have appeared in The Hospital on the opening for private nurses in Rangoon. I think it only right that your readers should know how hopeless in most cases it would be for a nurse to come out without a fixed engagement or a guarantee of constant employment to an expensive country without a friend. The climate is very damp and enervating, though hardly unhealthy enough to offer brilliant prospects for private nurses. My experience has been that the only work that keeps nurses in employment is midwifery. Some excellent nurses have already established their practice there and got a connection. They have been able, no doubt, to secure constant employment, and probably one of these is the nurse to whom your lady correspondent alludes. But even these nurses do not depend on Rangoon alone: their practice extends to Upper Burmah—I may say truthfully, hundreds of miles off. The station hospitals provide for the nursing of military officers at the rate of 2 rupees per diem inclusive. If the officers had a private nurse they would have her and themselves to keep, stimulants to provide, and the nurse to pay from 5 to 10 rupees per day. Civilians can be nursed in private wards in the civil hospitals. A number of ladies for their confinements go now to the Dufferin Hospital, where they can be nursed and have medical attendance in private wards for 4 rupees per diem. One lady doctor takes private cases into her own house. The civil hospitals and the Dufferin, in Burma, are all training nurses, and the Karen women trained at the Dufferin are excellent nurses and go out at a much cheaper rate than the European or Eurasian nurses. They cost less to board, do not need the same amount of waiting on as the others, and are therefore much preferred by many, and are infinitely more useful than English nurses would be fresh out from home, as they know the ways and can speak the language of the servants. Living is very expensive in Rangoon, and the distances are very great. It is a large town, and introductions to medical men are necessary. There are no lodgings to be had as in England. The nurse would require three servants at least: a boy, who would act as housemaid and cook, at about £1 6s. 8d. a month a sweeper, who would sweep her rooms and attend to the sanitary arrangements: he or she would cost 6s. 8d. a month. There are no sanitary conveniences as in England. All filth and rubbish and dirty water have to be carried by sweeper to conservancy receptacles, and they are carted away at night.

The cost of necessary utensils at the outset could not be got under £1. The cost of living at a hotel or boarding house is from 10s. 8d. to 12s. 8d. per day. Three nurses living in Rangoon, apart from house rent, furniture, etc., found it cost them, living as cheaply as they could, 198 rs. per month, or £13 4s. a month. As one nurse would need the same number of servants, the cost for her would be £7 15s. Another nurse I knew took what she thought two very cheap rooms: they were £4 a month for rent only. I also knew a nurse who came out as stewardess: she could not get employed, so worked her passage back. I am thankful to say that I have left Burmah, and am therefore an unprejudiced witness.

### A DIFFICULTY IN NURSING.

"B. M. C." writes from one of the Colonies: I wonder if any of my sister nurses have experienced difficulties such as I met with in one of my cases. I can laugh at them now, but they were none the less real at the time. My patient was a dear little seven months' old baby, suffering from dysentery. For a time its condition gave us great anxiety, but I am glad to say the little one was spared, and I left it looking well and healthy. My troubles were not connected with the baby but with its mother who was an ardent, but entirely inexperienced advocate of homœopathy; she had little or no faith in allopathy, and would, if she could, have called in a homœopathic doctor. There was none, however, within reach, and the despised allopath was summoned. Then my worries began. The doctor—a good little man—prescribed certain treatment, and I essayed to carry out his instructions; he did not know then, though I fancy he guessed later, what difficulties I worked under. The mother seemed to regard me with suspicion, and seemed perfectly certain that doctor and I were in league to injure her precious child. No food nor medicine of the doctor's ordering was looked on with favour, and the cold glances of the mother were decidedly disconcerting. Baby did not improve immediately, whereupon the poor woman grew desperate and appealed to me and my natural kindness of heart (she evidently doubted much I had any!) to make some change in our treatment. She descanted on the merits of homœopathic drugs; recommended chammonilla, pulsatilla, bryonia, and such like, but in this I was adamant, and refused to experiment without the doctor's orders. The appealing look turned again to one of coldness and distrust, and for several days I worked with the pleasing consciousness that I was being regarded in the light of an unfeeling automaton. "You cannot understand my feelings, nurse," she said one day. This was an improvement; she was beginning to speak out, and I had got the opening I wanted. I tried to show her—poor anxious mother—that my training was acquired that I might assist doctors, not suggest or substitute on my own account other forms of treatment; I assured her I would do all that lay in my power to help her dear little baby, but that she, on her part, must try to have more faith in doctor and nurse. She listened patiently and after that she affected a demeanour that was even more trying than that of suspicion and scepticism. It was a martyr-like submission and resignation to the inevitable which was assuredly death for her baby. She even told me she was quite prepared for it, and that she would no longer interfere. She was consoled, too, by dosing herself abundantly with the despised homœopathic medicines, and asked me, on more than one occasion, to remind her at such and such an hour to take pulsatilla alternately with bryonia. This I was willing to do, but I could not help a rather wicked twinkle in my eye when I brought the matter to her mind at the appointed time. Her spare moments were often filled by dips into a musty and much used homœopathic book, and occasionally passages were read aloud for my edification. Finally, baby got better, and its mother and I became very good friends; but all her talking failed (much to her sorrow) to turn me from the error of my allopathic ways and convert me to homœopathy, and I left her bemoaning the fact, and declaring that if only I would go in seriously for homœopathy, I should be a much greater success in my profession than hitherto, and of far greater service to mankind in general.

### Appointments.

[No charge is made for announcements under this head, and we are always glad to receive, and publish, appointments. But it is essential that in all cases the school of training should be given.]

Bromsgrove Union.—Miss Ellen Annie Sims has been appointed superintendent nurse. She was trained at Stapleton Infirmary, Bristol, where she was afterwards charge nurse.

Dewsbury Workhouse Infirmary.—Miss Meta Jackson has been appointed charge nurse. She was trained at Chorlton Union Infirmary, West Didsbury, Manchester.

Hitchin Workhouse Infirmary.—Miss Fanny Buxton has been appointed superintendent nurse. She was trained at the Queen's Hospital, Birmingham, for three years, and has since been nurse at Monsall Fever Hospital, Manchester; Southwark Infirmary, East Dulwich; Croydon Infirmary, and Oulton Union Infirmary, Lowestoft, where she was superintendent.

New Annexes, Tooting Home, S.W.—Miss Florence M. Boyce has been appointed sister in charge. She was trained at the North London Hospital for Consumption and Diseases of the Chest, and also for three years at the Mile End Infirmary, where she was staff nurse for a year. She has since been attached to the All Saints Nursing Institution, and has done private nursing; and has lately been engaged as lecturer on nursing to the London School Board, Victoria Embankment, W.C.

Northampton Borough Hospital, Kingsthorpe.—Miss M. C. Tarrant has been appointed head nurse. She was trained at the North Staffordshire Infirmary and Eye Hospital, Stoke-on-Trent, for three years, and has since been charge nurse at the South Western Fever Hospital, Stockwell. She has also done private nursing at Eastbourne and London.

Sir Patrick Dun's Hospital, Dublin. — Miss L. V. Haughton has been appointed lady superintendent. She was trained at Guy's Hospital, and has since been sister of "Patience" ward, and sister of "Astley Cooper" ward.

St. Catherine's Cottage Hospital, Clun, Salop.—Miss Margaret Sleap has been appointed matron. She was trained at Leeds Hospital for Women and Children and Chichester General Infirmary, and has since held appointments at the West Kent General Hospital, Maidstone; the Cathedral Nurses' Home, Newcastle-on-Tyne; the Metropolitan Convalescent Institution, Walton-on-Thames; and district nurse, West Hartlepool.

St. Leonard's Infirmary, Shoreditch.—Miss Ellen Fleury has been appointed ward sister. She was trained at Camberwell Infirmary for three years, and was previously assistant nurse at Stepney Union Infirmary and the Brook Hospital, London.

St. Thomas's Hospital.—Miss Harriette E. G. Hamilton has been appointed matron. She was trained at St. Thomas's Hospital, where she was afterwards sister. She has since been matron of Carlisle Infirmary, matron of the Victoria Hospital for Children, Chelsea, and matron of University College Hospital, London.

Tunbridge Wells General Hospital.—Miss Elizabeth Keith has been appointed matron. She was trained in the Western Infirmary, Glasgow, and has been for a year night sister in the General Hospital, Colchester, and for 18 months assistant matron in the City of London Hospital for Diseases of the Chest.

Wolstanton and Burslem Union Infirmary.—Miss Clara Page has been appointed superintendent nurse. She was trained at Leeds Union Infirmary, and has had fever training at Wolverhampton Borough Hospital. She has since been assistant nurse at Birmingham Eye Hospital, charge nurse at Kendal General Hospital, head nurse at Bradford City Hospital, matron at Morecambe Fever Hospital, and charge sister at Birmingham City Hospital.

### TRAVEL NOTES AND QUERIES.

BRUGES (All things to those who wait).—Thanks for your interesting notes. There is an article about to come out on the subject. We do not put correspondents in communication with each other, but I shall be much obliged for the address if you will kindly send it to me. Board, with very moderate terms, is a perpetual want. I fancy from your description it may be the Convent of the Sacré Cœur, which I know well; all personal experience of hotels, boarding houses, etc. . . . I am most grateful for, as it is impossible to try them all myself.

EXCURSIONS FROM PERROS-GUIREC (Randolph).—From Perros-Guirec you can visit Lannion. The journey is by diligence, and only costs 75 centimes, and from there be sure to see Ploumanac'h and Tregastel. Paimpol is another place to see by diligence, and Treguier. I am not sure if there is a public conveyance to this, but I think so. I went by carriage. Treguier is inland and very pretty. If you can, a few days might be spent at Morlaix, one of the most interesting towns in Brittany. Go to the Hôtel de l'Europe or the Hôtel Bozellec. Ask for terms, on the third or fourth floor: it is not a cheap place, but very delightful. From there visit Roscoff, St. Pol de Lebon. Also you might take train to Huelgoat, 20 miles to the south: it is not remarkable scenery, but it gives you the idea of Central Brittany scenery.

THE CHANNEL ISLANDS (Country Woman).—You will not find it so cheap as Belgium, but very charming. Go to Guernsey and Sark; the latter is quite delightful. Second return to Guernsey £2 5s. 6d., third return £1 18s. The cheapest hotel at Guernsey that I know of is the Crown, 5s. 6d. per day; if you cannot get in there try the Broughton, 6s. 6d. These are in St. Peter Port, the chief town. I think it would be worth your while to go into the town and see for lodgings, of which doubtless there are plenty. There is a restaurant, Taylor's, where you can dine if it is more convenient. The same may be said of Sark, the hotels Bel Air and the Dixcart, but I know there are lodgings. Steamer from St. Peter Port four times a week. You can go for the day only, fares 2s. 6d. You would not care, I think, to stay in Alderney. You can visit it from Guernsey; inquire hours and dates at Guernsey itself. If you go to the Channel Islands you will find Black's 1s. guide useful. With regard to the Scilly Isles, I do not think them very suitable for a short holiday; the chief ones are St. Mary, Tresco, and St. Martin. When the weather is bad communication with the mainland is cut off, which is awkward. I should not advise it. However these are the hotels at St. Mary's: In Hugh Town, Tregarthen's; at Tresco, The Canteen; at St. Martin's there is no inn. Let me know if I can help you further.

HOLIDAY FOR THREE NURSES (K. B.).—We do not insert such advertisements, and there is no charge for answering questions in this section. Paris is generally dear, but try what the three can be taken for at this address: M. Ledoux, 20 Rue Clairant, Avenue de Clichy, Batignolles; also very reasonabl ethe Hôtel Britannique, 20 Avenue Victoria; also Mme. Mansfield, 157 Faubourg, Saint-Honoré. I have not used this last address very lately, and Mme. M. may have moved; if still there, it is a most comfortable place. I think, if there are three of you, and out of the season, you would be treated very liberally. How would you like Brittany? Mme. Pallot, Maison Mathias, St. Servan, Ille-et-Vilaine, will take you for 6 or 7 francs per day; three together probably for less. About the same terms at Miss Humphrey's, 20 Place Constantine, St. Servan. At Brussels you would find it very pleasant and gay. Write for terms to the Hôtel du Rhin, 14 Rue de Brabant; or the Hôtel Royal, Boulevard du Hainault; or the Hôtel Croisades. I don't remember the street, but the name is enough. These cheaper hotels are often moved, and one does not hear of it; so write in good time. Terms about 6 francs per day, or less for three. Tell me if I can help further, and next time please give a pseudonym as well as your own.

### To Nurses.

**WE invite contributions from any of our readers, and shall be glad to pay for "Notes on News from the Nursing World," or for articles describing nursing experiences, or dealing with any nursing question from an original point of view. The minimum payment for contributions is 5s., but we welcome interesting contributions of a column, or a page, in length. It may be added that notices of appointments, entertainments, presentations, and deaths are not paid for, but that we are always glad to receive them. All rejected manuscripts are returned in due course, and all payments for manuscripts used are made as early as possible after the beginning of each quarter.**

### For Reading to the Sick.

### LIFE'S LESSONS.

THIS life is but a school-time,
In which we learn to love
The friends we see around us,
The unseen God above.

Some learn by active service,
Others, in grief and pain;
Some seem to reap in gladness,
The rest, to toil in vain.

The great thing is, to study
To seek our Lord in all:
His great Love to remember,
Whatever may befall.

And pain and weakness make Him
Nearer and dearer seem,
Till life becomes a story
Of which He is the theme.

*C. M. Noel.*

Nothing is too little to be ordered by our Father; nothing too little in which to see His hand; nothing, which touches our souls, too little to accept from Him; nothing too little to be done to Him.—*E. B. Pusey.*

. . . Wherever souls are being tried and ripened, in whatever commonplace and homely ways;—there God is hewing out the pillars for His temple. Oh, if the stone can only have some vision of the temple of which it is to lie a part forever, what patience must fill it as it feels the blows of the hammer, and knows that success for it is simply to let itself be wrought into what shape the Master wills.

*Phillips Brooks.*

The noble love of Jesus impels a man to do great things, and stirs him up to be always longing for what is more perfect.—*Thos. à Kempis.*

He has kept and folded us from ten thousand ills when we did not know it; in the midst of our security we should have perished every hour, but that He sheltered us "from the terror by night and from the arrow that flieth by day"—from the powers of evil that walk in darkness, from snares of our own evil will. He has kept us even against ourselves, and saved us even from our own undoing. Let us read the traces of His hand in all our ways, in all the events, the chances, the changes of this troubled state. It is He that folds and feeds us, that makes us to go in and out—to be faint, or to find pasture—to lie down by the still waters, or to walk by the way that is parched and desert.—*H. E. Manning.*

"For I have loved thee with a love
No mortal heart can show;
A love so deep, My Saints in heaven
Its depths can never know:
Vain are thy offerings, vain thy sighs,
Without one gift Divine,
Give it, My child, thy heart, to Me,
And it shall rest in Mine!" . . .—*A. A. P.*

### Echoes from the Outside World.

### The Coronation.

It was officially announced on Saturday that, by the King's command, the coronation of their Majesties will take place on Saturday, August 9th. The Queen has written to Colonel Gildea, of the Soldiers' and Sailors' Families Association, saying that she will attend the annual meeting of that organisation on Friday, August 8th. The route of the Coronation procession followed will be the same as that arranged for June 26th, and so far as the seats or stands erected by the Government are concerned, all holders of tickets which were issued in June are entitled to places now. But it is requisite to apply for new tickets, all the old ones having been cancelled. A pleasing evidence of the King's progress is that the ambulance carriage used for his removal has been returned to store.

### Royal Sympathy for Suffering.

The King and Queen occupy some of the time which they now have at their disposal in doing kindnesses to those of their subjects whom they know to be suffering from distress of body or mind. A little boy of ten, a solo chorister at Holy Trinity Church, Beckenham, underwent an operation at Guy's Hospital for perityphlitis on the same day as the King. Peritonitis set in and he died. The case was made known to Queen Alexandra who sent the following letter through one of her private secretaries to the mother: "Buckingham Palace.—Madam,—The Queen has heard of the great loss which you have sustained by the death of your little boy. I am commanded to express her deep sympathy with you and your husband." A resident of Chatham who appealed to her Majesty for help has not gone unaided. The poor woman pleaded that her husband was away and that she was in poverty and trouble. The Queen caused full inquiries to be made, and then sent her correspondent a present of £3 in money besides gifts in kind, in the way of tea, coffee and cocoa. An old lady, living at Middlesbrough, has just received a message from the King, sympathising with her in her illness and hoping that she will recover. The old lady was the daughter of a soldier and married a private, with whom she went through two Kaffir wars. She served ammunition in the trenches at Fort Hare, and assisted in the nursing of the wounded, for whom her wedding outfit was sacrificed to make bandages. Her father and brother were murdered by Kaffirs while eating their Christmas dinner. All Mrs. Hirst's five sons entered the army, three rose to be captains, one a bandmaster, and the fifth a sergeant of the Medical Corps. Mrs. Hirst's only daughter married a soldier, and has two sons serving the King. Lord Roberts brought the case of the old lady to the King's notice.

### Politics and Society.

Lord Cadogan has resigned the office of Lord-Lieutenant of Ireland, and on Friday he informed his Cambridgeshire tenants that he thought the retirement from the Premiership by Lord Salisbury seemed to afford him an occasion on which it would be becoming for him to discontinue the labours in which he had for so long been engaged. Lord Cadogan is 64 years of age, and has held several important official positions, including those of Under-Secretary for War, Under-Secretary for the Colonies, and Lord Privy Seal. While he was Viceroy of Ireland he entertained the present Prince and Princess of Wales on two occasions, but the visit of Queen Victoria to Ireland in 1900 was the most important event that happened during his term of office.

The death of Mr. J. W. Mackay, known both in English and American society as "the Silver King," took place at 6 Carlton House Terrace on Sunday evening. He was taken ill suddenly in the City on the previous Tuesday, and his condition gradually became worse. Mr. Mackay, who was a native of Dublin, began life in humble circumstances, but, emigrating to America, he acquired wealth rapidly in the Californian goldfields. In 1872 he and others discovered the famous Bonanza lode, which in four years paid a profit of over 15 millions sterling. Mr. Mackay, who was the president of the Commercial Cable Company, had for some years past resided principally in Europe. His adopted daughter is now Princess Ferdinand de Colonna, and was present when he died; but his son, Mr. Clarence Mackay, is now on his way to England.

### The Boers.

The repatriation of the Boers has already started. Four hundred men arrived from Colombo at Durban last week on their way home. Some of those left behind in Ceylon have been giving trouble because they refused to believe that peace had been proclaimed, and the riot resulted in twenty of the Boer prisoners and some of the Gloucesters being injured. An Englishman who has had an opportunity of conversing with Christian De Wet and his military adviser, Commandant Schultz, was much impressed with their proficiency in the English language. In common with the rest of the Boer leaders De Wet appeared quite resigned to the new order of things and determined to be on good terms with the English. He said that "he had come to the conclusion, during the war, that if the English and Dutch would combine as men and brothers they could defy the whole world." Commandant Botha and ex-Commandant Delarey have left Pretoria on their road to the Cape *en route* for Europe. They are to be joined later by De Wet.

### St. Mark's, Venice.

Active measures are being taken to rebuild the Campanile, or belfry of St. Mark's, Venice, the fall of which, last week, is looked upon by all lovers of the beautiful in art and architecture as a great disaster. The accident is said to be due to the lightning stroke which the tower received in 1745, though some think that the foundations, which have been laid for over a thousand years, have crumbled away. The inhabitants of Venice were so overcome at the collapse of their beautiful tower that they wept, and between £20,000 and £30,000 was subscribed the day after the accident, it being also proposed that a manifesto appealing for funds should be issued to all the artists in the world. Before, however, this could be done, a cable from New York from Count Morrisini offering to contribute £20,000 towards the building made such an appeal unnecessary. The bronze gates have been found intact, also the whole front cornice with the columns and angels, and the beautiful bronze of Mercury has been recovered and is only damaged in the hands. But statues, bronzes, and marbles could almost all have been saved if the precaution of removing them had been taken when first the cracks were noticed. The fall was fortunately unattended with loss of life, though no attempt was made even to warn people away from the Square of St. Mark. Luigi Vendrasco made a report on the Campanile in 1892, saying it was badly in need of repair, but no notice was taken of his warning.

### Savings Bank Funds.

It appears from the report of the Select Committee of the House of Commons, appointed to inquire into the Savings Bank Funds, that already the ratepayers, during the last 25 years, have been obliged to find over £500,000 to provide the necessary interest on deposits, the money earned by the savings of the thrifty not being sufficient to maintain the rate of interest promised. In view of the fact that after next year the interest on Consols will be reduced from $2\frac{3}{4}$ to $2\frac{1}{2}$ per cent., and most of the Savings Bank Funds are invested in Consols, it has been determined to lower the interest offered to depositors by $\frac{1}{8}$ per cent., because otherwise the loss in 1903 would be £136,000, and the amount having to be made up by taxation. An entry in a depositor's pass-book is after this year to be a legal receipt without any further acknowledgment from the General Post Office, and neither the trustee nor the Post Office Savings Bank shall any longer be obliged to send depositors a notice when their account reaches £200. These apparently trifling alterations will effect a saving of £33,000 a year.

### Notes and Queries.

**The Editor is always willing to answer in this column, without any fee, all reasonable questions, as soon as possible.**

**But the following rules must be carefully observed :—**

**1. Every communication must be accompanied by the name and address of the writer.**

**2. The question must always bear upon nursing, directly or indirectly.**

**If an answer is required by letter a fee of half-a-crown must be enclosed with the note containing the inquiry, and we cannot undertake to forward letters addressed to correspondents making inquiries. It is therefore requested that our readers will not enclose either a stamp or a stamped envelope.**

*Home.*

(127) Can you kindly tell me of a home for a lady of 55, who has been a national school teacher. She is willing to be useful, and is musical, but is subject to occasional attacks of epilepsy. Friends could pay 10s. a week for her board ?—*Interested.*

See reply to Queen's Nurse.

Can you tell me of a home for a lady needing constant supervision. I think that drink is possibly the cause of the trouble. She could afford to pay about 14s. weekly ?—*Toby.*

You might write to the Secretary, the British Women's Temperance Association, 47 Victoria Street, London, S.W.

I should feel so much obliged if you could kindly tell me of a home where a gentleman of 80 suffering from paralysis in its worst form could receive every comfort. We are willing to pay three or four guineas a week, and would like a place near this address if possible.—*I. C.* (Bowdon, Cheshire).

We do not give recommendations, but there are plenty of private nursing homes which would receive the patient at the terms you mention.

1. Can you kindly tell me of a convalescent home for a woman in Lancashire where invalid ways are more or less allowed ? 2. Can you give me any remedy for corns on the sole of my foot ?—*A. M.*

1. The Southport Convalescent Hospital and Sea Bathing Infirmary might meet your requirements. 2. Consult a medical man.

1. Can you inform me of a home where an epileptic young woman could be received free or nearly so ? 2. Will you kindly insert the following advertisement ?—*Queen's Nurse.*

1. The Meath Home of Comfort for Epileptics, Westbrook, Godalming, seems the best for your purpose. 2. Send advertisement to the Manager.

*Hospital Training.*

(128) Will you kindly give me names and addresses of any hospitals where by payment one could get three months' practical training in general nursing ?—*A. M. T.*

Many hospitals offer facilities of this kind. Consult the "Nursing Profession : How and Where to Train."

I am 19, strong and healthy, and am anxious to become a hospital nurse when I am old enough. Will you kindly tell me what subjects I should take up and what books I should read ?—*M. B.*

Study all domestic arts as all knowledge comes in handy to a nurse. Read improving books on subjects congenial to you, and if you have any gift for music, cultivate it, for when your nursing studies begin you will have little time for any other. A well-stored mind is useful not only to the nurse, but to the patients who will be dependant on her for companionship through hours of sickness and convalescence.

Is it usual for a House Committee of a cottage hospital to give certificates for nursing efficiency ? Is it proper to give a certificate without the recipient having attended courses of lectures and having passed an examination by the staff or selected examiners ?—*Enquirer.*

Unless the hospital is recognised by the Local Government Board as a training school for nurses, the certificates granted are of no value in the nursing world.

I am assistant matron at a boys' orphanage and would like to become a nurse, will you kindly tell me what qualifications are necessary ?—*Stoke Newington.*

You must obtain a three years' certificate from a recognised training school. All information, together with a full list of training schools, will be found in "The Nursing Profession : How and Where to Train."

I am anxious to become a hospital nurse, but will the fact of both parents having died of consumption prevent my being accepted for training ?—*F. B.*

Probably. Why not try some outdoor employment such as gardening ?

I should like to be a hospital nurse, but as I have had to undergo two operations for uterus, I should be glad if you will tell me if that will prevent my being trained.—*Burley.*

Nurses ought to be unusually strong to enable them to stand the hard work of their calling. We are afraid that you do not answer this description.

*Massage.*

(129) I am a certificated masseuse, and I should be very glad if you could tell me of any agency which would inform me of the best means of obtaining work —*L. K.*

The Secretary of the Society of Trained Masseuses, 12 Buckingham Street, Strand, W.C., would doubtless be able to help you.

Will you kindly tell me the best place to study massage. I do not wish to go to great expense. Is not Norway the best ?—*M. M. C.*

Will you kindly tell me where I could get a good course of instruction in massage, and also tell me what the fee would be ?—*Aspirant.*

The National Hospital for the Paralysed and Epileptic, Queen's Square, Bloomsbury, is as good and as cheap as any.

I do not belong to the Society of Trained Masseuses. Do you think it would be to my advantage to do so ?—*M. M. K.*

Yes, it is an advantage to keep in touch with other members of the same craft, besides, the Secretary of the Society of Trained Masseuses is always ready to advise on difficult cases, and to recommend patients. As only masseuses of good training and character are put on the register, it is itself a recommendation to belong to it.

Will you kindly tell me if there is a thoroughly good teacher of massage residing at Manchester ?—*Agnes.*

Apply to the Secretary of the Society of Trained Masseuses.

*Market Nurse.*

(130) Having heard that there has been a nurse appointed to Billingsgate Market, I should like to know if there are any similar posts to be obtained, and what are the requirements ?—*E. P. B.*

Such appointments are likely to be filled up locally, and your only chance of securing one is by answering an advertisement if you should happen to come across it.

*Dispenser.*

(131) Will you kindly tell me if there is a decent living to be made as lady dispenser ? I am 20 years of age, well educated, and am anxious to know.—*I. C. G. and K. R. A. O.*

There is an opening for well-qualified women. The training is rather expensive and difficult. See the "Englishwoman's Year Book" for particulars.

Would you kindly tell me where I could get the cheapest instruction in dispensing, and say if it would be necessary to understand Latin.—*M. F. V.*

Apply to the Secretary, the Pharmaceutical Society, 17 Bloomsbury Square, W.C. Some little knowledge of Latin is needed.

*Pathologist.*

(132) I am an assistant in a private laboratory, and have plenty of spare time. Do you think I could get a few hours' work daily at a hospital or laboratory as pathologist's assistant ? I have had plenty of experience.—*Microbe.*

You can find out by advertising in the medical journals.

*Beef Juice.*

(133) Mince or cut finely 1 lb. of beef. Add half a pint of water and one or two drops of hydrochloric acid (this draws out all the essence of the beef), stand it in a cool place until drawn, then add one part claret to four parts beef juice, and serve in a claret glass. So many patients object to the beef juice alone, and it is both tempting and palatable served in this way.—*M. M. K.*

*Sleep.*

(134) Is it injurious for a patient to sleep with her arms over her head ?—*Nurse Mary.*

Not, if the arms and chest are kept warm.

In which position is it best to lie, in order that one may sleep well ? Does it make any difference which point of the compass one faces, and if so why ?—*Paddie.*

It has been thought that it is best to have the head of the bed placed towards the north, in order that the body may lie parallel with the magnetic currents of the earth. There is no doubt that intensely nervous patients might be benefited by this if they were firmly convinced of its efficacy. The position most recommended is to sleep on the back, slightly turned on the right side.

**Standard Nursing Manuals.**

"The Nursing Profession : How and Where to Train." 2s. net ; post free 2s. 4d.

"Art of Massage." (Second Edition.) 6s.

"Elementary Physiology for Nurses." 2s.

"Elementary Anatomy and Surgery for Nurses." 2s. 6d.

"Practical Handbook of Midwifery." 6s.

"Surgical Ward Work and Nursing." Revised Edition. 3s. 6d. net ; post free 3s. 10d.

"Mental Nursing." 1s.

"Art of Feeding the Invalid." 1s. 6d.

All these are published by the Scientific Press, Ltd , and may be obtained through any bookseller or direct from the publisher, 28 and 29 Southampton Street, London, W.C.

### Travel Notes.

By Our Travelling Correspondent.

### CIV.—WHAT WE CAN SEE FOR FIVE POUNDS.

### Caudebec, in Normandy.

There are not very many places in Brittany and Normandy that one can visit for a week and spend only £5; or perhaps it would be more correct to say that though there are several, it is imperative that they should not be far distant from the disembarkation points to save the cost of railways. This fact rather circumscribes our wanderings. If only one need not consider much the expense of getting there, in lower Brittany there are delightful places, ideally cheap, in which to spend a holiday, but reaching them is both long in time and hard on one's purse. Trains in Brittany are leisurely and it requires great determination and energy to cover the ground between north and south in one day, though the distance as the crow flies is not more than 80 to 100 miles between the extremest points. Another day I hope to speak a little of these more distant places to those who have three weeks or a month at their disposal.

### The Journey to Caudebec-en-Caux.

Second-class return to Candebec itself will be £1 18s. 9d., but if you are cyclists you might like to run up from Havre on your machines, which would lessen the expense to £1 11s. 8d. From July to September steamers run from Havre to Rouen every day and *vice versâ*, and you would be dropped at Caudebec into the hospitable embrace of the Hôtel de la Marine, which is actually on the quay, and which saves considerably in tips, etc. Here you will live in comfort and luxury for 7 francs per day (5s. 10d.), or even less by arrangement.

If you are cycling you can push on to Caudebec easily from Havre, where you arrive in the early morning. You will take with you what things you imperatively need for the night, and send the others on by post. If you only intend to spend a week in France your impedimenta need be very slight. On your route you can see Harfleur, Tancerville, and Lillebonne. Harfleur is a quaint old town, chiefly interesting from the fact of our conquest of it under our Henry V. There is a fine church, and still many curious and picturesque co-ners to delight the eye of the searcher after the artistic. You will pass through Tancerville, worth seeing on account of its massive feudal castle; and Lillebonne, where you had better lunch frugally at the Hôtel du Commerce, reaching Caudebec in comfortable time that evening.

### The Town Itself is pleasing and old world, full of quaint half-timbered houses and unexpected nooks, with delightfully rich-hued houses at all kinds of angles overhanging back waterways. Baedeker hardly condescends to notice the existence of modest Caudebec, and naturally in such a voluminous work as his, many a charming spot must perforce be disregarded, or his valuable books would want a library all to themselves, and of no limited dimensions either. I have, in my wanderings, found many an entrancing unnoticed corner of the earth, quite unmentioned in the pages of our honoured guides, Messrs. Baedeker, Black, Johanne, etc., and in consequence of their immunity from the chronicler, they still retain a simplicity and a charm, fast disappearing before the all conquering knapsack of the tourist. The church (fifteenth and sixteenth centuries) possesses a very magnificent west front; I believe it is not architecturally correct to lavish such admiration on it as I do, but I am never weary of studying its beauties and marvelling over the reverent patience that carved those doors, buttresses, and parapet.

The interior resembles somewhat St. Jacques in Dieppe, with its numerous side chapels. Observe the magnificent roof of the Lady Chapel, with its tremendous carved pendant. I do not know the correct name for this, and will not therefore draw down on me the derision of the erudite by using an incorrect term.

### St. Wandrille and Jumiéges.

A short walk (not more than two miles) is to St. Wandrille. The monastery originally built in the seventh century was rebuilt in the fourteenth, and extensively restored in 1863 by an Englishman who purchased it. There is still a good deal left of the original construction, notably a part of the cloisters and refectory. There is, too, in the village, a parish church of considerable interest.

Jumiéges must ever be an important spot to lovers of romance; it is easily reached from Caudebec by rail, river, or road. It is entrancing to stand within the grass-grown precincts and remember the generations that once worshipped there and have for ever passed away from this world, whilst kindly time has cast its misty veil over the erring and the virtuous alike. Our thoughts naturally turn to the frail but lovable Agnes Sorrel, who will surely be forgiven much, for she loved much. She lived near by at Mesnilsous-Jumiéges at one time, and there she died, and was buried in the Abbey. On her tombstone it is recorded that she was "Pieteuse aux pauvres." Her heart only reposed here, her body, by order of her royal lover, being removed to Loches.

The first sight of Jumiéges with its splendid twin towers is most impressive; our Romsey Abbey is somewhat in the same style, though considerably smaller. The oldest untouched part of Jumiéges dates from 1067, and the effect of alternate square and round columns is grand and pleasing too.

Inside all is in a state of ruin, no roof and the pavement has been long covered by grass, but I much prefer it in this state of decay to seeing it blossom (however well done) into a spurious and youthful perfection under the hand of the ardent restorer.

### Excursions round Caudebec.

These are endless. A diligence starts daily from your little hotel to Yvetôt, 8 miles to the north, the drive takes you through pretty country, and there are some interesting houses in the town. I think it was here that "Madame Bovary" lived part of her by no means blameless life, and found it so "ennuyant," which indeed I can well believe to a lady of her temperament. You will of course visit La Bouille, it is a great resort of artists, and I must bow to their superior opinion, but I prefer Moulineux which is most charmingly placed on the river, and has a fine church. Then there are St. Georges-de-Boscherville-Duclair and many other delightful spots, all easily attainable.

---

Rules in Regard to Correspondence for this Section.—All questioners must use a pseudonym for publication, but the communication must also bear the writer's own name and address as well, which will be regarded as confidential. All such communications to be addressed "Travel Editor, 'Nursing Section of *The Hospital*,' 28 Southampton Street, Strand." No charge will be made for inserting and answering questions in the inquiry column, and all will be answered in rotation as space permits. If an answer by letter is required, a stamped and addressed envelope must be enclosed, together with 2s. 6d., which fee will be devoted to the objects of "The Hospital" Convalescent Fund. Any inquiries reaching the office after Monday cannot be answered in "The Hospital" of the current week.

## Figures and Tables

**Fig. 51. f1:**
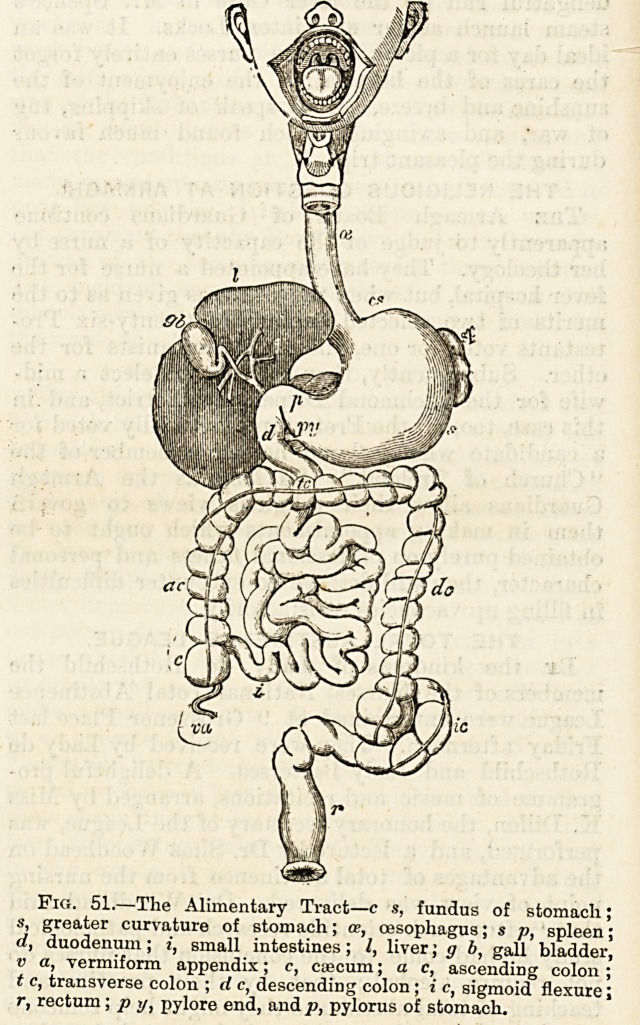


**Fig. 52. f2:**